# A Biobrick Library for Cloning Custom Eukaryotic Plasmids

**DOI:** 10.1371/journal.pone.0023685

**Published:** 2011-08-25

**Authors:** Marco Constante, Raik Grünberg, Mark Isalan

**Affiliations:** EMBL/CRG Systems Biology Research Unit, Centre for Genomic Regulation (CRG) and UPF, Barcelona, Spain; Deutsches Krebsforschungszentrum, Germany

## Abstract

Researchers often require customised variations of plasmids that are not commercially available. Here we demonstrate the applicability and versatility of standard synthetic biological parts (biobricks) to build custom plasmids. For this purpose we have built a collection of 52 parts that include multiple cloning sites (MCS) and common protein tags, protein reporters and selection markers, amongst others. Importantly, most of the parts are designed in a format to allow fusions that maintain the reading frame. We illustrate the collection by building several model contructs, including concatemers of protein binding-site motifs, and a variety of plasmids for eukaryotic stable cloning and chromosomal insertion. For example, in 3 biobrick iterations, we make a cerulean-reporter plasmid for cloning fluorescent protein fusions. Furthermore, we use the collection to implement a recombinase-mediated DNA insertion (RMDI), allowing chromosomal site-directed exchange of genes. By making one recipient stable cell line, many standardised cell lines can subsequently be generated, by fluorescent fusion-gene exchange. We propose that this biobrick collection may be distributed peer-to-peer as a stand-alone library, in addition to its distribution through the Registry of Standard Biological Parts (http://partsregistry.org/).

## Introduction

The construction of specific plasmid DNA sequences is a routine technique in molecular biology laboratories [1]. The most widely used DNA sequences (for instance, a promoter followed by a fluorescent protein, a multiple cloning site and a polyadenylation signal) are easily found in commercially-available plasmids [2], [3]. However, when requirements start to become more stringent, multiple plasmid modifications are required and, although many changes may be relatively simple to perform, multiple modifications may become time-consuming cloning challenges.

**Figure 1:**
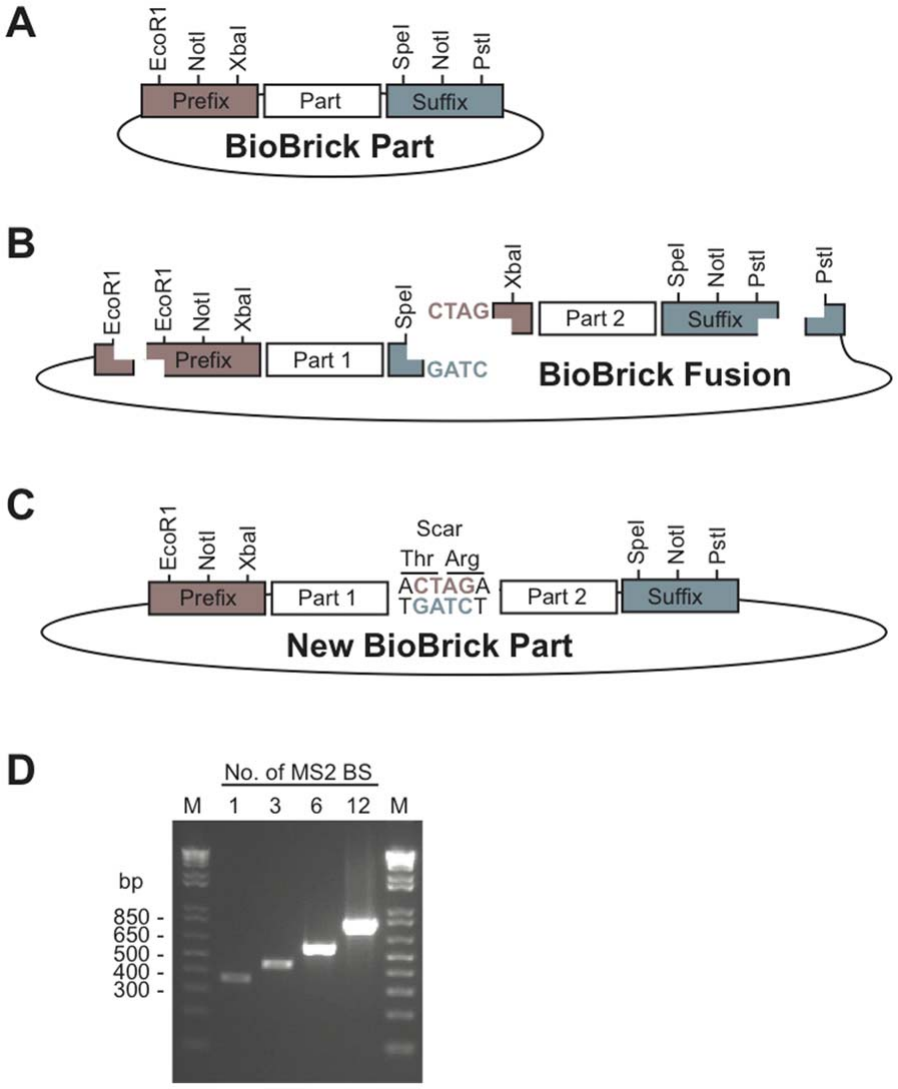
The biobrick assembly principle[8], [10]. (A) Each biobrick part has the same prefix and suffix, containing restriction enzyme sites. (B) Following restriction digests, a two-insert ligation into the biobrick vector results in a biobrick fusion. (C) The new biobrick part regenerates the original prefix and suffix, but contains an in-frame Thr-Arg scar in protein-coding fusions. (D) MS2 binding site concatemers (MS2 BS), built with iterative biobrick assembly, from 1 to 12-copies (4 steps). M = marker (1 kb ladder). The upstream and downstream sequences between the primer annealing sites and the biobricks contribute 312 bp, while each MS2 BS is 39 bp.

Complex, multi-factor plasmids have to be built frequently for the applications of synthetic biology [4], [5], [6], often with the combinatorial use of different DNA parts [7]. To simplify such types of constructions an idempotent cloning system has recently been developed by Tom Knight [8] (described in the BioBrick Foundation Request for Comments #10, BBF RFC 10; http://biobricks.org/). Briefly, this system uses a specific set of restriction enzyme sites at the 3′ and 5′ ends of each DNA cassette (‘biobrick’), such that a biobrick ‘A’ may be fused with a biobrick ‘B’ to produce ‘AB’. ‘AB’ contains an uncleavable ‘scar’ sequence between ‘A’ and ‘B’ and, importantly, the exact same set of restriction enzyme sites as the initial biobricks, at the 3′ and 5′ ends. In other words, every biobrick fusion product is itself a new biobrick and may even be used iteratively for the assembly of concatemers (See Figure 1). Given the physical idempotent characteristics of the system, biobricks may be fused together in any combination of parts, with few restrictions in the number of the biobricks, and no restrictions on the order (‘BA’ would be as simple to construct as ‘AB’).

The RFC 10 Biobrick format [8] is itself very useful and has formed the core of engineering challenges such as the annual International Genetically Engineered Machine (iGEM) competitions [9], where students are asked to engineer systems using biobricks. It is a well-documented system with a large and growing collection of parts that use the prefix GAATTCGCGGCCGCTTCTAGAG (or GAATTCGCGGCCGCTTCTAG for protein coding parts starting with ATG) and the suffix TACTAGTAGCGGCCGCTGCAG. However, the original scheme has certain limitations, such as the difficulty of fusing parts coding for protein domains, since the fusion ‘scar’ changes the codon reading frame from biobrick ‘A’ to biobrick ‘B’. Phillips and Silver [10] resolved this issue by presenting a slight modification to the initial design (removing the last G of the prefix and the first T of the suffix thereby conserving the reading frame in the scar). Special attention is required when preparing new parts in this format (BBF RFC 23), since the removal of the last G of the prefix means that a Dam methylation site may be formed if the part starts with TC, which blocks restriction with Xba I. Since the scar resulting from a RFC 23 fusion (ACTAGA) codes for threonine-arginine, a chimeric protein ‘AB’ may be assembled from two RFC 23 biobricks ‘A’ and B’. When preparing such RFC 23 protein fusions, users should be aware that the arginine from the scar may be problematic since it is positively charged.

It is of interest to note that biobricks in the RFC 23 format may still be assembled with biobricks in the RFC 10 format without requiring new restriction enzymes. Although such a RFC 23-RFC 10 cross-fusion does not maintain the 6-bp scar required for the maintenance of a codon frame, N-terminal fusions of RFC 23 protein coding parts may still be performed as long as the frameshift is corrected by an adaptor part. In addition, the RFC 23-RFC 10 cross-fusion may be useful for assemblies that do not require the maintenance of a reading frame or a specific scar size, such as linking non-coding biobricks (e.g. transcription factor binding sites). We refer to this compatibility between RFC 23 and RFC 10 as ‘assembly compatibility’, that should not be confused with ‘RFC 23 compatibility’ that is reserved for fusion between biobricks that maintain the reading frame. Further biobrick formats (with only limited cross-compability) were later proposed and are documented as BBF RFCs.

Engineering with the Biobrick system is usually accompanied with an abstraction hierarchy perspective. With this perspective in mind, one uses ‘DNA’ to design basic ‘parts’ that may then be assembled into a single functional ‘device’ with a specific function. Finally, several of these ‘devices’ may be put together to design a synthetic ‘system’ with a high-level functionality. This hierarchy facilitates synthetic engineering since, ideally, a researcher working, for instance, at the ‘system’ level does not need to know the specific details on how to engineer and build the ‘devices’ used in that ‘system’.

Synthetic biologists are now in the process of developing and characterizing basic ‘parts’ or part sets (‘devices’ [11]) that may be used to engineer biological machines [12], [13], [14] and to model them [15]. These parts are available from the Registry of Standard Biological Parts (http://partsregistry.org/) which is supported by the BioBricks Foundation (http://bbf.openwetware.org/).

Although the registry is available to any researcher, whether or not they work in the field of synthetic biology, we believe that the size of the registry, the existence of different biobrick formats (not always compatible with each other), and the limited communication of this resource to non-synthetic biologists, may hamper the evolution and distribution of biobricks. We therefore developed a cloning tool that should be of interest outside the field of synthetic biology; a biobrick toolkit for the construction of custom eukaryotic expression plasmids, using frequently-used components. We originally noted that many biobrick parts that one would require for eukaryotic projects were apparently missing from the registry. These parts included: several multiple cloning sites with different reading frames; an extensive range of reporter proteins; eukaryotic selection markers; eukaryotic internal ribosomal entry sites; and protein epitope tags. Importantly, most of these parts should be compatible with each other [10], [16]. In addition, the novel use of MCS biobricks facilitates the cloning of DNA fragments of interest (e.g. open reading frames, promoters), without having to first remove any internal cleavage sites of the biobrick enzymes in order to render them ‘biobrick compatible’.

This work is intended to highlight the potential of the biobrick system to construct custom plasmids for eukaryotic cell lines, for both synthetic and non-synthetic biologists. Since many parts that we consider important did not exist in the registry, we have built a distribution of 9 previously existing and 43 new biobricks. We demonstrate the utility of the new biobricks with some examples of assemblies. In addition, we use the created library to establish a gene-switching system, from tdTomato to EGFP expression, using recombinase-mediated DNA insertion (RMDI). Finally, we propose that this collection be distributed peer-to-peer as an independent library, as well as being available from the registry, and discuss some advantages in having this type of collection.

## Materials and Methods

### Preparation of parts

New parts were designed following the recommendations in BBF RFC 23. Briefly, parts were formatted to contain the prefix 5′-GAATTCGCGGCCGCTTCTAGA-3′ and the suffix 5′-ACTAGTAGCGGCCGCTGCAG-3′. Construction of the parts was done using 1) oligonucleotide inserts, 2) PCR with oligonucleotide-directed mutagenesis when required [1] or 3) synthesized directly by GenScript Corporation (Piscataway, NJ). Importantly, the part sequences do not start with a TC since this forms a Dam methylase site (GATC) and digestion with XbaI can be inhibited. The Biobrick sequences are available at the Registry of Standard Biological Parts website (http://partsregistry.org/), at GenBank (http://www.ncbi.nlm.nih.gov/genbank/) and in the Supporting Information S1.

### Biobrick assemblies

Assemblies were performed similarly to the method presented in the Biobrick assembly kit from New England Biolabs (NEB; Ref. E0546S) and the Gingko Bioworks manual (http://ginkgobioworks.com/support/). Alternatively, one may use the adapted streamlined protocol we have previously reported [14].

Briefly, 500 ng of the upstream part are digested with 20000 U of EcoRI (NEB) and 10000 U of SpeI (NEB) in NEB EcoRI buffer. At the same time, 500 ng of the downstream part are digested with 20000 U of XbaI (NEB) and 20000 U of PstI (NEB) in NEB Buffer 3. Finally and concomitantly, 500 ng of the destination plasmid are digested with 20000 U of EcoRI and 20000 U of PstI in NEB EcoRI Buffer. Digestions are performed at 37°C for 1 hour followed by heat inactivation at 80°C for 20 minutes. Note that the recipient pSB1A* plasmids in the library contain a *ccdB* death cassette that is removed in the digestion. Furthermore, each assembly should contain an antibiotic resistance gene that is not present in either the upstream or the downstream part (e.g. if the upstream part is in the pSB1AK3 plasmid, containing ampicillin and kanamycin resistance, and the downstream part is in the pSB1AC3 plasmid, containing ampicillin and chloramphenicol resistance, the destination plasmid should be the pSB1AT3 plasmid since it contains the tetracycline resistance not present in the other plasmids).

Since the destination plasmid contains the *ccdB* death cassette, a two-way ligation of the fragments can be done using the Roche rapid DNA ligation kit, according to the manufacturer's instructions, without need for gel isolation or other purifications. Briefly, 4 µl of the upstream part digestion, 4 µl of the downstream part digestion and 2 µl of the destination plasmid digestion are used. 2 µl of the ligation product are transformed in one shot competent Top10 *E. coli* (Invitrogen), according to the manufacturer's instructions, and are subsequently plated onto LB agar plates. The plates contain antibiotic for the resistance provided by the destination plasmid (in the example above one should use LB agar plates containing tetracycline) and are incubated overnight.

PCRs of Biobricks (from plasmid DNA or bacterial colony) were performed using NEB Taq polymerase, using the manufacturer's instructions, and using the standard sequencing primers BBa_G00100 (5′-TGCCACCTGACGTCTAAGAA-3′) and BBa_G00101 (5′-ATTACCGCCTTTGAGTGAGC-3′) with 20 cycles at 55°C annealing temperature, with appropriate denaturation and elongation steps. PCR products were analyzed on agarose gels and positive colonies were grown overnight for DNA isolation using the Qiagen spin miniprep kit according to the manufacturer's instructions. All clones were verified by DNA sequencing.

### Cell culture and transfection

HEK293 cells were purchased from ATCC. Cells were propagated in Dulbecco's Modified Eagle Medium (Gibco) supplemented with 10% fetal bovine serum (Gibco) and 1% penicillin-streptomycin (Gibco). Transfection of the cells was performed with lipofectamine 2000 (Invitrogen), according to the manufacturer's instructions, in 6-well plates: cells were incubated for 4 hours with 4 µg of DNA and 10 µl of lipofectamine reagent. Stable clones were prepared by selection with 0.5 mg/ml G418 (Sigma).

### Cloning small Espin into the MCS of a custom-made plasmid

Rat small Espin (sEspin) [17] was amplified by PCR, from a plasmid kindly provided by the group of Hernán López-Schier, using the primers sEspinF (5′-AAGAGGGATCCATGAACTCCC-3′) and sEspinR (5′-CTTCTTACCGGTTTACTTAGGGATCTCCCCCTTC-3′) at 55°C annealing temperature. The PCR product was cloned into the Biobrick custom plasmid using the restriction enzymes BamHI and AgeI.

### Microscopy

After transfection, cells were allowed to recover overnight and were dispensed into glass bottom culture dishes (MatTek). 24 h later, the medium was replaced with PBS for observation under the microscope.

### Recombinase-mediated DNA inclusion plasmid preparation

The plasmid used for the creation of the stable clone expressing tdTomato was prepared using an assembly of the biobricks between the CMV promoter and polyadenylation (pA) signal. The assembled plasmid was digested with XbaI and PstI and cloned into pEGFP-C1 digested with NheI and PstI, thereby removing the EGFP sequence and providing the CMV upstream and pA downstream of the biobrick insert. Neomycin resistance used for selection is provided by the pEGFP-C1 plasmid backbone under the expression of the SV40 early promoter. The EGFP plasmid for inclusion was prepared using the biobrick collection.

### Flow cytometry

Flow-assisted cell sorting (FACS) analysis was performed with FACSCanto (BD) and cell separation with FACSARIA II (BD).

## Results

### Library

**Figure 2:**
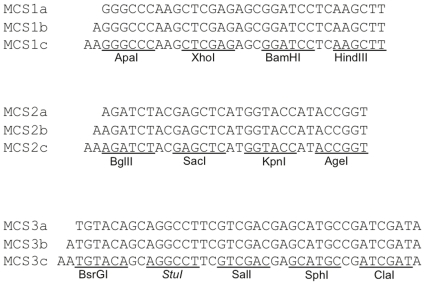
Multiple Cloning Site (MCS) biobricks. The uniqueness of each cloning site is dependent on whether the remaining biobricks and backbones used for the custom plasmid also contain the restriction site. Blunt end restriction enzymes are represented in italic. These biobricks link classical cloning to the biobrick system.

To create a distribution for the construction of custom plasmids, we have put together a set of 9 existing and 43 new biobricks and plasmid backbones. We believe that these should be useful for the creation of custom-made plasmids for use in eukaryotic cell lines (see Table 1). Importantly, a set of multiple cloning site (MCS) biobricks, covering all three different frames has been built, essential for the combination of the biobrick assembly strategy with classical cloning strategies (Fig. 2).

**Table 1:** List of biobrick parts for eukaryotic plasmids.

Group	Nickname	Description	Registry	GenBank
Backbones	pSB1A3*	High copy number plasmid carrying ampicillin resistance.	pSB1A3	
	pSB1AK3*	High copy number plasmid carrying ampicillin and kanamycin resistance.	pSB1AK3	
	pSB1AC3*	High copy number plasmid carrying ampicillin and chloramphenicol resistance.	pSB1AC3	
	pSB1AT3*	High copy number plasmid carrying ampicillin and tetracyclin resistance.	pSB1AT3	
Construction	Kozak	Simple Kozak sequence protein head domain [26]	BBa_J96000	JN204869
	Stop	Tail domain with stop codons in all three frames	BBa_J96001	JN204870
	CMV*	Cytomegalovirus immediate-early promoter	BBa_I712004	
	SV40pA*	Eukaryotic – derived from SV40 early poly A signal sequence	BBa_J52016	
	MCS1a	Multiple cloning site version 1, first frame	BBa_J96002	JN204871
	MCS1b	Multiple cloning site version 1, second frame	BBa_J96003	JN204872
	MCS1c	Multiple cloning site version 1, third frame	BBa_J96004	JN204873
	MCS2a	Multiple cloning site version 2, first frame	BBa_J96005	JN204874
	MCS2b	Multiple cloning site version 2, second frame	BBa_J96006	JN204875
	MCS2c	Multiple cloning site version 2, third frame	BBa_J96007	JN204876
	MCS3a	Multiple cloning site version 3, first frame	BBa_J96008	JN204877
	MCS3b	Multiple cloning site version 3, second frame	BBa_J96009	JN204878
	MCS3c	Multiple cloning site version 3, third frame	BBa_J96010	JN204879
Selection	FNeomycin	Resistance to G418/neomycin gene [1]	BBa_J96011	JN204880
	FPuromycin	Resistance to puromycin gene [1]	BBa_J96012	JN204881
	FHSTK	Herpes simplex thymidine kinase conferring toxicity to ganciclovir [27]	BBa_J96013	JN204882
Reporters	tdTomato	Engineered red fluorescent protein [28]	BBa_J96029	JN204883
	EGFP	Engineered green fluorescent protein [29]	BBa_J96031	JN204884
	Cerulean	Engineered cyan fluorescent protein [19]	BBa_J96032	JN204885
	EBFP2	Engineered blue fluorescent protein [30]	BBa_J96033	JN204886
	mCherry*	Engineered red fluorescent protein [28]	BBa_J63000	
	RLuciferase	Renilla luciferase gene [31]	BBa_J96034	JN204887
Tags	Flag	FLAG affinity tag [32]	BBa_J96035	JN204888
	HA	HA affinity tag [33]	BBa_J96036	JN204889
	His	His affinity tag [34]	BBa_J96037	JN204890
	StrepII	Strep II affinity tag [35]	BBa_J96038	JN204891
Localization	SP	Membrane or secretion: IgK leader peptide with Kozak [36]	BBa_J96014	JN204892
	TMD	PDGF Receptor Transmembrane Domain [36]	BBa_J96015	JN204893
	Myristoylation	Myristoylation signal sequence with Kozak [37]	BBa_J96016	JN204894
	NLS*	Nuclear Localization Signal from SV40 [38]	BBa_J63008	
Recombination	Loxp	Lox p sequence [39]	BBa_J96017	JN204895
	Lox66	Directional lox sequence compatible with lox71 [40]	BBa_J96018	JN204896
	Lox71	Directional lox sequence compatible with lox66 [40]	BBa_J96019	JN204897
Cistron	IRES	Internal ribosomal entry site [41]	BBa_J96040	JN204898
	P2A	Self cleaving 2A peptide [42]	BBa_J96041	JN204899
	T2A	Self cleaving 2A peptide [42]	BBa_J96042	JN204900
	PS3	Short DNA sequence for ribosome recruitment (mini-IRES) [43]	BBa_J96043	JN204901
	PS4	Short DNA sequence for ribosome recruitment (mini-IRES) [43]	BBa_J96044	JN204902
Others	Linker	24 aa flexible linker, rich in Gly and Ser.	BBa_J96020	JN204903
	Spacer1*	Randomized DNA spacer	BBa_J96021	
	Spacer2	Randomized DNA spacer	BBa_J96022	JN204904
	MS2	MS2 phage coat domain binding to RNA at the MS2 binding site sequence [44]	BBa_J96023	JN204905
	MS2BS	MS2 phage coat binding site sequence [44]	BBa_J96024	JN204906
	LambdaN	Lambda N peptide sequence binding to RNA at the boxB binding site [45]	BBa_J96025	JN204907
	BoxB	Lambda N peptide binding site sequence [45]	BBa_J96026	JN204908
	TEVSite	TEV tobacco etch virus protease cleavage site [46]	BBa_J96027	JN204909
	d1PEST	Mouse ornithine decarboxylase PEST sequence 1 hr half-life [47]	BBa_96046	JN204910
	d2PEST	Mouse ornithine decarboxylase PEST sequence 2 hr half-life [47]	BBa_96047	JN204911

Asterisks (*) indicate previously existing Biobricks. “Registry” refers to the Registry reference, whereas “GenBank” refers to the GenBank accession number.

To facilitate flexible construction of custom-made plasmids, all biobricks are in the same format, containing the same prefix and suffix, as described in the Methods section. Biobricks may be divided into: (i) Non-coding (not intended for containing coding sequences; lengths are often not in multiples of three, or have a stop codons in the sequence). (ii) Head domains (that contain a Kozak sequence and are used to start the translation of the protein of interest). (iii) Internal domains (the coding regions for the desired domains (e.g. reporter proteins). (iv) Tail domains (containing the stop codon used to stop the translation of the protein of interest). (v) Translation units (containing the Kozak sequence, an internal domain and a stop codon). Table 2 indicates whether a given biobrick maintains the codon frame, whether it contains a Kozak sequence or a stop codon, and shows the restriction sites present in each biobrick or plasmid backbone. In this table we explicitly present those elements that may not be used for biofusion assembly (highlighted in bold).

**Table 2:** Characteristics of the collection plasmids.

	Name	Restriction Sites	Kozak	Stop	Backbone	Size(bp)	3n	Well
Backbones	**pSB1A3**		-	-	-	2157*	-	1A
	**pSB1AK3**	ClaI, HindIII, SmaI, XhoI	-	-	-	3189*	-	1B
	**pSB1AC3**	SacI, XhoI	-	-	-	3055*	-	1C
	**pSB1AT3**	BamHI, ClaI, HindIII, SalI, SphI, XhoI	-	-	-	3446*	-	1D
Construction	Kozak		YES	N	AK	12	YES	1E
	**Stop**		N	**YES**	AK	11	N	1F
	**CMV**	SacI	N	**YES**	AK	654	YES	1G
	**SV40pA**	SphI	N	**YES**	AK	228	YES	1H
	**MCS1a**	ApaI, XhoI, BamHI, HindIII	N	N	AK	32	N	2A
	MCS1b	ApaI, XhoI, BamHI, HindIII	N	N	AT	33	YES	2B
	**MCS1c**	ApaI, XhoI, BamHI, HindIII	N	N	AT	34	N	2C
	MCS2a	BglII, SacI, KpnI, AgeI	N	N	AT	30	YES	2D
	**MCS2b**	BglII, SacI, KpnI, AgeI	N	N	AT	31	N	2E
	**MCS2c**	BglII, SacI, KpnI, AgeI	N	N	AT	32	N	2F
	MCS3a	BsrGI, StuI, SalI, SphI, ClaI	N	N	AC	39	YES	2G
	**MCS3b**	BsrGI, StuI, SalI, SphI, ClaI	N	N	AC	40	N	2H
	**MCS3c**	BsrGI, StuI, SalI, SphI, ClaI	N	YES	AC	41	N	3A
Selection	**FNeomycin**	SphI	YES	**YES**	AK	801	YES	4A
	**FPuromycin**	StuI	YES	**YES**	AC	606	YES	4B
	**FHSTK**	ApaI, SmaI, SphI	YES	**YES**	AC	1137	YES	4C
Reporters	tdTomato		N	N	AK	1425	YES	5A
	EGFP	BsrGI	N	N	AC	714	YES	5B
	mCerulean	BsrGI, ClaI	N	N	AC	714	YES	5C
	EBFP2		N	N	AC	714	YES	5D
	mCherry		N	N	A	705	YES	5E
	RLuciferase	BsrGI, SphI	N	N	AK	930	YES	5F
Tags	Flag		N	N	AC	24	YES	6A
	HA		N	N	AC	27	YES	6B
	His		N	N	AC	18	YES	6C
	StrepII		N	N	AC	24	YES	6D
Localization	SP		YES	N	AC	69	YES	7A
	TMD		N	N	AC	147	YES	7B
	Myrist		YES	N	AC	48	YES	7C
	NLS		N	N	A	21	YES	7D
Recombination	Loxp		N	N	AC	36	YES	8A
	Lox66		N	N	AC	36	YES	8B
	Lox71		N	N	AC	36	YES	8C
Cistron	**IRES**	ApaI, HindIII, KpnI	N	**YES**	AC	504	YES	9A
	P2A	SmaI	N	N	AC	42	YES	9B
	T2A		N	N	AC	54	YES	9C
	**PS3**	BsrGI	N	**YES**	AC	50	N	9D
	**PS4**		N	**YES**	AC	48	YES	9E
Others	Linker		N	N	AC	72	YES	10A
	**Spacer1**		N	**YES**	A	72	YES	10B
	**Spacer2**		N	N	AK	70	N	10C
	MS2	BglII, SacI, SalI	N	N	AK	387	YES	10D
	MS2BS	SalI	N	N	AC	39	YES	10E
	LambdaN		N	N	AC	66	YES	10F
	BoxB	ApaI	N	N	AC	21	YES	10G
	TEVSite		N	N	AC	21	YES	10H
	**d1PEST**		N	**YES**	AT	129	YES	11A
	**d2PEST**		N	**YES**	AT	129	YES	11B

Characteristics of the collection plasmids. In bold we present all plasmids that, when fused, do not allow for a continuous coding sequence (either because they contain a stop signal or because the length in bp is not a multiple of 3: “3n”). A (ampicillin), C (chloramphenicol), K (kanamycin) and T (tetracycline) indicate the resistances provided by each backbone. The position of samples in the distribution is indicated by “Well”. Asterisks (*) indicate that the presented size refers to the plasmid size instead of the biobrick size.

Whereas the biobrick assembly workflow will work for all parts in the collection, for classical cloning it is up to the user to check which biobricks will be used to assemble a plasmid. For example, the user must check whether a given restriction enzyme site is present only once (e.g. in the MCS site region) or whether it is not unique and is also found in another biobrick or plasmid backbone.

### Constructing simple assemblies

The biobrick assembly principle is inherently iterative, allowing longer and longer poly-biobrick constructs to be built in a stepwise fashion (Fig. 1). This is particularly useful for making related constructs with variable copies of a motif, such as a transcription factor binding sites. To illustrate this, we show how the binding sites of the bacterial MS2 phage coat protein, which can be used to repress mRNA translation in synthetic biology applications [18], can be conveniently concatenated with biobrick assembly (Fig. 1D).

**Figure 3:**
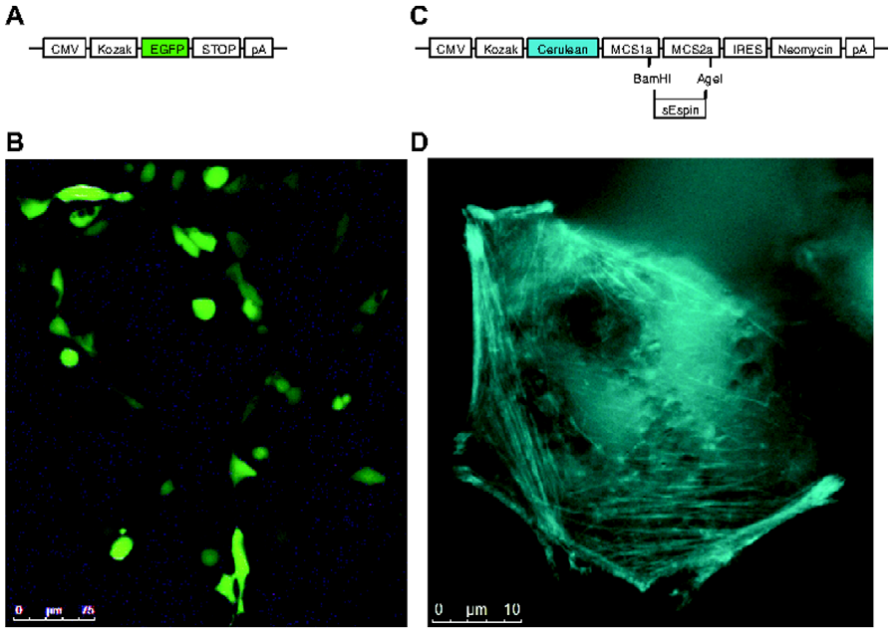
Examples of biobrick assemblies. (A) Structure of a classical plasmid for EGFP expression using 5 biobricks. (B) Structure of bicistronic custom plasmid. 8 biobricks are linked together to make a construct for C-terminal fusions to the blue fluorescent protein, cerulean, using classical restriction enzyme multiple cloning sites (MCS). For illustration, the actin-bundling binding protein sEspin is cloned into the MCS, resulting in a fusion with cerulean. (C) Fluorescence microscopy image of the EGFP construct in panel A, after transient transfection into HEK293 cells. (D) A fluorescence microscopy image of the Cerulean-sEspin fusion construct in panel B, allows the visualisation of stress fiber-like structures in a HEK293T cell. Scale bars are indicated below each image.

One of the simplest expression plasmids that one might construct using the distribution is a plasmid such as that presented in Figure 3A; a simple EGFP expression plasmid, used to label cells, as may be observed in Figure 3B. A user will typically want to create a translation unit and insert it under a promoter with a polyadenylation sequence in the end. The translation unit will typically be composed of a head domain (in this case the Kozak sequence alone) followed by one or more internal domains (in this example the EGFP reporter coding sequence) and finished with a tail domain (here the simple stop codon biobrick).

### Constructing a MCS plasmid for classical cloning

Although many commercially-available plasmids contain MCSs to allow fusing proteins of interest to fluorescent proteins, many combinations of colours, N-C orientations, stable cell-line selection genes or half-life modifications are simply not available. To illustrate this we have created a more complex plasmid, containing two cistrons, that is not commercially-available. In the first cistron, we assembled the blue fluorescent protein cerulean [19] before a complex MCS (assembled using MCS1 and MCS2). We subsequently cloned an actin-binding protein, rat sEspin [17], using the BamHI and AgeI restriction sites (Figure 3C). By fluorescent microscopy, we observed Cerulean-sEspin localization in actin bundle stress fiber-like structures [17] (Fig. 3D). In the second cistron we included the neomycin resistance gene for positive selection. The example shown is just one of many customized cloning vectors that can be generated with this platform, and requires just three biobrick cloning iterations (first round: CMV-Kozak, Cerulean-MCS1a, MCS2a-IRES, Neomycin-pA; second round: CMV-Kozak-Cerulean-MCS1a, MCS2a-IRES-Neomycin-pA; third round: CMV-Kozak-Cerulean-MCS1a-MCS2a-IRES-Neomycin-pA). The use of MCSs links the biobrick format to classical cloning strategies, which should increase their appeal to researchers who simply wish to ‘cut and paste’ their DNA cassettes with standard restriction enzymes.

### Recombinase-Mediated DNA Insertion

**Figure 4:**
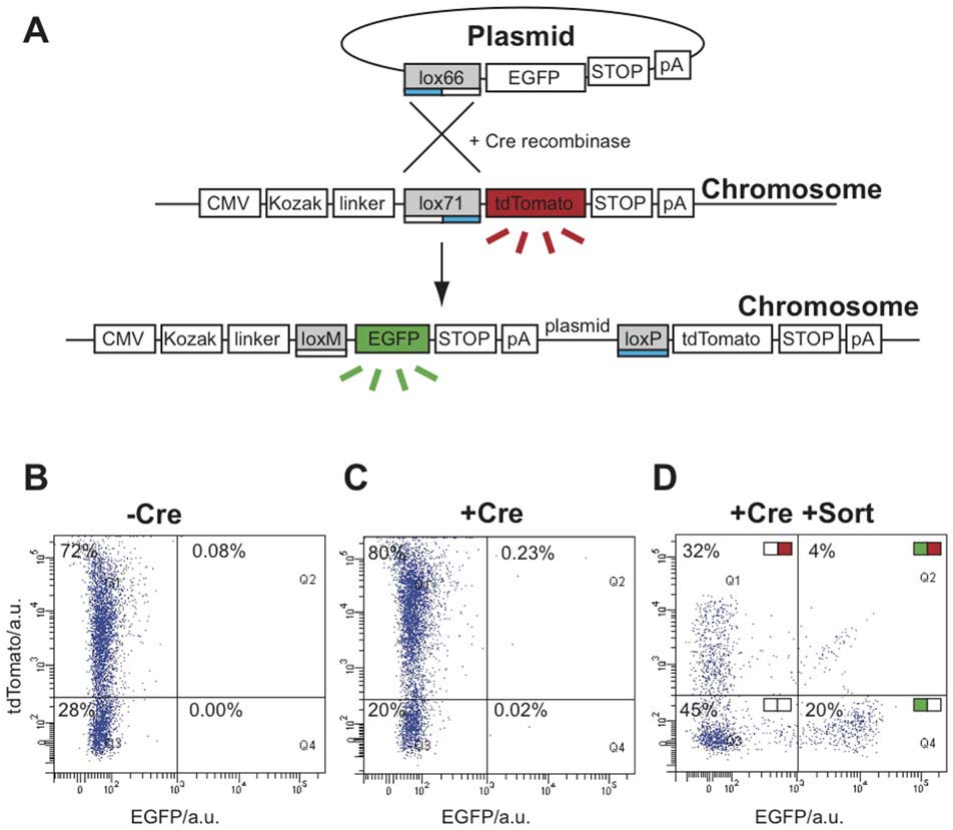
Recombinase-mediated DNA insertion with biobricks. (A) Schematic view of Cre-mediated recombination between lox66 and lox71, resulting in the insertion of EGFP and creating a mutated lox site (loxM) and a loxP site. This prevents the original tdTomato from being expressed while allowing EGFP expression. (B, C and D) Flow cytometry analysis of a stable cell line expressing tdTomato, with various recombinase or cell sorting treatments. (B) The untreated cell line (−Cre) contains mainly red-positive cells (72%). (C) Upon recombinase treatment (+Cre), the amount of green cells increases in quadrants 2 and 4 (Q2, Q4). (D) By sorting the green cells (Q2 and Q4 in the previous panel), using flow cytometry (+Sort), and growing to confluency, the resulting cells are enriched for the newly-generated green cells (20%; Q4).

The collection presented here may be used to obtain stable cloning systems, for chromosomal integration in eukaryotic cells, such as Recombinase-Mediated DNA Insertion (RMDI) [20]. We implemented RMDI by establishing a stable cell line expressing tdTomato, and containing an heteromeric lox site in its coding sequence (lox66 [21]; see scheme in Figure 4A). Through Cre-mediated recombination we inserted DNA from a donor plasmid, containing EGFP preceded by a compatible heteromeric site (lox71). Thus, red expression was converted to green expression.

Since the EGFP plasmid did not contain a promoter, cells should express EGFP alone only if inserted at the correct RMDI site. Expression from random insertions next to genomic promoters (expected to be rare events) would be associated with both green and red expression. However, in the early stages after transfection, even correctly recombined cells would be expected to have some residual red expression, until the levels of tdTomato were degraded or diluted by cell division.

Indeed, after three days we observed a cell population expressing both EGFP and tdTomato by flow cytometry (0.23% for RMDI when using both the EGFP plasmid and Cre versus 0.08% for random insertion in the control with the EGFP plasmid without Cre; Fig. 4B and C). Thus, there appeared to be some Cre-induced increase in GFP fluorescence, but it was not clear whether the effects were site-specific or due to background fluorescence. Therefore, to investigate whether the tdTomato levels would subsequently fall, indicating correct RMDI, we sorted the EGFP-positive population in Figure 4C, grew them to confluency and re-analysed the final population. We thus obtained a population of cells where RMDI had indeed occurred, with high levels of EGFP and low levels of tdTomato, similar to non-fluorescent controls (20% green-only cells; Fig. 4D).

When considering only the EGFP-positive population (Q2+Q4) we note a marked shift in the Q4 population (8% of Q2+Q4 before sorting to 83% of Q2+Q4 after sorting and regrowth). This suggests that the insertion of the EGFP plasmid occurred at the intended lox site, thereby disrupting tdTomato, but that extra time was required for tdTomato levels to fall, via dilution and degradation, during regrowth of the sorted cells.

The 17% of Q2+Q4 found in Q2 is an EGFP-tdTomato double-positive population (4% of the total cell population; Fig. 4D), likely arising from cells containing multiple chromosomal copies of the initial tdTomato construct: here recombination occurred in one or more instances, but not in every copy. Random integrations of GFP near promoter regions are likely to be rare but may also contribute to this population.

We also observed tdTomato-positive EGFP-negative (32%) and tdTomato-EGFP double-negative (45%) populations. These are likely arising from cells that were unmodified and yet were carried-through the EGFP cell sorting step (false positives) or from cells that downregulated the CMV promoter [22].

## Discussion

In this work, we have created a distribution of DNA fragments compatible with the Standard Registry of Biological Parts [8]. This distribution is intended for the specific use of creating custom-made plasmids, with a focus on use in eukaryotic cell lines, particularly because many previous parts have focused on prokaryotic components. We have therefore constructed biobricks for elements that are routinely used in plasmids for transfection in eukaryotic cell lines. Using these elements, we provide three examples of applications of this system: the assembly of sequence motif concatemers; the assembly of vectors for transient and stable transfection; and a Cre-based Recombinase-Mediated DNA Insertion.

Although the present work makes use of existing concepts, such as the standardization of DNA parts [23] and classical cloning for expression in eukaryotic cell lines [1], the construction of the required elements into one collection and the combination of both cloning concepts makes this method a distinct new resource for preparing eukaryotic contructs. To provide this tool to users, we propose that the collection presented here be available as a stand-alone biobrick distribution exchanged in a peer-to-peer fashion, in addition to its availability through the Registry of Standard Biological Parts (http://partsregistry.org/).

A researcher may request access to the Registry to obtain a large collection of biobricks which are a valuable resource, especially to synthetic biologists. However, the size of the Registry database may be detrimental in some instances, especially for sporadic users. One example of this is the impossibility - at the moment - to search the registry database for biobricks in a specific format. In fact, special care must be taken when using biobricks from the registry since it is not always clear whether two internal protein domain biobricks may be fused in frame, because of the different biobrick formats [10], [16]. This is in part due to the definition of the registry database of biobricks as being ‘compatible’ with a given format when meaning they may be assembled together even if the coding frame is not maintained, whereas other users may consider ‘compatibility’ as an indication that the fusion conserves the codon frame.

In contrast, RFC 23 compatibility in the small collection presented here – in the sense of codon reading frame conservation – is ensured except where otherwise explicitly stated. Another advantage of having an independent small distribution for a specific function is the possibility to exchange it in 96-well plates and to be sure that one is using sequence-verified components. For example, when sequence-verifying the registry, certain discrepancies have been found [24]. The bacteria containing the plasmids can be grown and shipped in wells filled with agar medium, allowing direct sharing between peers, without need for advanced robotics or pipetting systems.

We believe that other similar biobrick libraries should be created to be self-contained. That is, all parts in such libraries should be compatible and sufficient to be used for a specific function. For instance one could envisage kinase-phosphatase or signalling cascade libraries; transcription factor libraries; metabolic enzyme libraries. Such a library-based form of distribution may be one of several solutions for the growing size of the Registry that may rapidly reach a limit in the cost-efficiency of its own distribution (the registry is >13 000 parts and growing [25]). Ultimately, we hope to stimulate debate in this growing open standard for biological engineering.

## Supporting Information

Supporting Information S1 **FASTA DNA sequences of the Biobrick Collection.** Prefix and Suffix are presented in capitals.
(DOC)
