# Supplementary material for: A Biobrick Library for Cloning Custom Eukaryotic Plasmids
Source: PLoS One. 2011 Aug 25;6(8):e23685. doi: 10.1371/journal.pone.0023685 (PMC3161993; doi:10.1371/journal.pone.0023685)
Supplement: Supporting Information S1 — FASTA DNA sequences of the Biobrick Collection. Prefix and Suffix are presented in capitals. (DOC) [file pone.0023685.s001.doc]

**Supporting Information**

FASTA sequences of Biobrick Collection. Prefix and Suffix are presented in capitals.

>BBa_J96000 Kozak Biobrick

GAATTCGCGGCCGCTTCTAGAgccaccatggttACTAGTAGCGGCCGCTGCAG

>BBa_J96001 Stop Biobrick

GAATTCGCGGCCGCTTCTAGAtagataactgaACTAGTAGCGGCCGCTGCAG

>BBa_I712004 CMV Biobrick

GAATTCGCGGCCGCTTCTAGAcgatgtacgggccagatatacgcgttgacattgattattgcctagttattaatagtaatcaattacggggtcattagttcatagcccatatatggagttccgcgttacataacttacggtaaatggcccgcctggctgaccgcccaacgacccccgcccattgacgtcaataatgacgtatgttcccatagtaacgccaatagggactttccattgacgtcaatgggtggagtatttacggtaaactgcccacttggcagtacatcaagtgtatcatatgccaagtacgccccctattgacgtcaatgacggtaaatggcccgcctggcattatgcccagtacatgaccttatgggactttcctacttggcagtacatctacgtattagtcatcgctattaccatggtgatgcggttttggcagtacatcaatgggcgtggatagcggtttgactcacggggatttccaagtctccaccccattgacgtcaatgggagtttgttttggcaccaaaatcaacgggactttccaaaatgtcgtaacaactccgccccattgacgcaaatgggcggtaggcgtgtacggtgggaggtctatataagcagagctctctggctaactagagaacccactgcttactggcttatcgaaatACTAGTAGCGGCCGCTGCAG

>BBa_J52016 SV40pA Biobrick

GAATTCGCGGCCGCTTCTAGActgtggaatgtgtgtcagttagggtgtggaaagtccccaggctccccagcaggcagaagtatgcaaagcatgcatctcaattagtcagcaaccaggtgtggaaagtccccaggctccccagcaggcagaagtatgcaaagcatgcatctcaattagtcagcaaccatagtcccgcccctaactccgcccatcccgcccctaactccgcccagttccgcccattctccgccccatggctgactaattttttttatttatgcagaggccgaggccgcctctgcctctgagctattccagaagtagtgaggaggcttttttggaggcctaggcttttgcaaaaagctcccgggagcttgtatatccattttcgACTAGTAGCGGCCGCTGCAG

>BBa_J96002 MCS1a Biobrick

GAATTCGCGGCCGCTTCTAGAgggcccaagctcgagagcggatcctcaagcttACTAGTAGCGGCCGCTGCAG

>BBa_J96003 MCS1b Biobrick

GAATTCGCGGCCGCTTCTAGAagggcccaagctcgagagcggatcctcaagcttACTAGTAGCGGCCGCTGCAG

>BBa_J96004 MCS1c Biobrick

GAATTCGCGGCCGCTTCTAGAaagggcccaagctcgagagcggatcctcaagcttACTAGTAGCGGCCGCTGCAG

>BBa_J96005 MCS2a Biobrick

GAATTCGCGGCCGCTTCTAGAagatctacgagctcatggtaccataccggtACTAGTAGCGGCCGCTGCAG

>BBa_J96006 MCS2b Biobrick

GAATTCGCGGCCGCTTCTAGAaagatctacgagctcatggtaccataccggtACTAGTAGCGGCCGCTGCAG

>BBa_J96007 MCS2c Biobrick

GAATTCGCGGCCGCTTCTAGAaaagatctacgagctcatggtaccataccggtACTAGTAGCGGCCGCTGCAG

>BBa_J96008 MCS3a Biobrick

GAATTCGCGGCCGCTTCTAGAtgtacagcaggccttcgtcgacgagcatgccgatcgataACTAGTAGCGGCCGCTGCAG

>BBa_J96009 MCS3b Biobrick

GAATTCGCGGCCGCTTCTAGAatgtacagcaggccttcgtcgacgagcatgccgatcgataACTAGTAGCGGCCGCTGCAG

>BBa_J96010 MCS3c Biobrick

GAATTCGCGGCCGCTTCTAGAaatgtacagcaggccttcgtcgacgagcatgccgatcgataACTAGTAGCGGCCGCTGCAG

>BBa_J96011 FNeomycin Biobrick

GAATTCGCGGCCGCTTCTAGAgccaccatgattgaacaagatggattgcacgcaggttctccggccgcttgggtggagaggctattcggctatgactgggcacaacagacaatcggctgctctgatgccgccgtgttccggctgtcagcgcaggggcgcccggttctttttgtcaagaccgacctgtccggtgccctgaatgaactgcaagacgaggcagcgcggctatcgtggctggccacgacgggcgttccttgcgcagctgtgctcgacgttgtcactgaagcgggaagggactggctgctattgggcgaagtgccggggcaggatctcctgtcatctcaccttgctcctgccgagaaagtatccatcatggctgatgcaatgcggcggctgcatacgcttgatccggctacctgcccattcgaccaccaagcgaaacatcgcatcgagcgagcacgtactcggatggaagccggtcttgtcgatcaggatgatctggacgaagagcatcaggggctcgcgccagccgaactgttcgccaggctcaaggcgagcatgcccgacggcgaggatctcgtcgtgacccatggcgatgcctgcttgccgaatatcatggtggaaaatggccgcttttctggattcatcgactgtggccggctgggtgtggcggaccgctatcaggacatagcgttggctacccgtgatattgctgaagagcttggcggcgaatgggctgaccgcttcctcgtgctttacggtatcgccgctcccgattcgcagcgcatcgccttctatcgccttcttgacgagttcttctaaACTAGTAGCGGCCGCTGCAG

>BBa_J96012 FPuromycin Biobrick

GAATTCGCGGCCGCTTCTAGAgccaccatgaccgagtacaagcccacggtgcgcctcgccacccgcgacgacgtccccagggccgtacgcaccctcgccgccgcgttcgccgactaccccgccacgcgccacaccgtcgatccggaccgccacatcgagcgggtcaccgagctgcaagaactcttcctcacgcgcgtcgggctcgacatcggcaaggtgtgggtcgcggacgacggcgccgcggtggcggtctggaccacgccggagagcgtcgaagcgggggcggtgttcgccgagatcggcccgcgcatggccgagttgagcggttcccggctggccgcgcagcaacagatggaaggcctcctggcgccgcaccggcccaaggagcccgcgtggttcctggccaccgtcggcgtctcgcccgaccaccagggcaagggtctgggcagcgccgtcgtgctccccggagtggaggcggccgagcgcgccggggtgcccgccttcctggagacctccgcgccccgcaacctccccttctacgagcggctcggcttcaccgtcaccgccgacgtcgagtgcccgaaggaccgcgcgacctggtgcatgacccgcaagcccggtgcctaaACTAGTAGCGGCCGCTGCAG

>BBa_J96013 FHSTK Biobrick

GAATTCGCGGCCGCTTCTAGAgccaccatggcttcgtacccctgccatcaacacgcgtctgcgttcgaccaggctgcgcgttctcgcggccatagcaaccgacgtacggcgttgcgccctcgccggcagcaagaagccacggaagtccgcctggagcagaaaatgcccacgctactgcgggtttatatagacggtcctcacgggatggggaaaaccaccaccacgcaactgctggtggccctgggttcgcgcgacgatatcgtctacgtacccgagccgatgacttactggcaggtgctgggggcttccgagacaatcgcgaacatctacaccacacaacaccgcctcgaccagggtgagatatcggccggggacgcggcggtggtaatgacaagcgcccagataacaatgggcatgccttatgccgtgaccgacgccgttctggctcctcatgtcgggggggaggctgggagttcacatgccccgcccccggccctcaccctcatcttcgaccgccatcccatcgccgccctcctgtgctacccggccgcgcgataccttatgggcagcatgaccccccaggccgtgctggcgttcgtggccctcatcccgccgaccttgcccggcacaaacatcgtgttgggggcccttccggaggacagacacatcgaccgcctggccaaacgccagcgccccggcgagcggcttgacctggctatgctggccgcgattcgccgcgtttacgggctgcttgccaatacggtgcggtatctccagggcggcgggtcgtggtgggaggattggggacagctttcggggacggccgtgccgccccagggtgccgagccccagagcaacgcgggcccacgaccccatatcggggacacgttatttaccctgtttcgggcccccgagttgctggcccccaacggcgacctgtataacgtgtttgcctgggccttggacgtcttggccaaacgcctccgtcccatgcacgtctttatcctggattacgaccaatcgcccgccggctgccgggacgccctgctgcaacttacctccgggatggtccagacccacgtcaccaccccaggctccataccgacgatctgcgacctggcgcgcacgtttgcccgggagatgggggaggctaactaaACTAGTAGCGGCCGCTGCAG

>BBa_J96029 tdTomato Biobrick

GAATTCGCGGCCGCTTCTAGAgtgtctaagggcgaggaagtgatcaaagagttcatgaggtttaaggtgagaatggaagggagtatgaacggccacgagttcgaaattgagggagaaggggaggggaggccctatgagggcacccagacagccaagctgaaagtgacaaagggcggacctctgccattcgcttgggacatcctgtctccacagtttatgtacggcagtaaggcttacgtgaaacatccagctgacattcccgattataagaaactgtctttccccgagggcttcaagtgggaaagagtgatgaacttcgaggacgggggcctggtgactgtgacccaggacagcagcctccaggatggaaccctgatctacaaggtgaaaatgagaggaacaaattttccccctgatgggcctgtgatgcagaagaaaactatggggtgggaggccagcaccgaacggctgtatccacgcgacggagtgctgaaaggggaaatccaccaggctctgaagctgaaagatggagggcattacctggtggagttcaagacaatctacatggccaagaaacctgtgcagctgccaggctactattacgtggacacaaaactggatatcacttctcacaacgaggactacactattgtggagcagtatgaaaggagtgagggaagacaccatctgttcctgggacatggaactgggagcaccggctccggatctagtgggaccgccagctccgaggataacaatatggctgtgatcaaagagttcatgcggtttaaggtgcgcatggagggcagcatgaatggacacgaatttgagattgaaggagagggagaaggacggccttatgagggcacacagactgccaagctgaaagtgaccaagggcggacccctgcctttcgcttgggatatcctgagccctcagtttatgtacgggtccaaggcttacgtcaagcatcccgctgacattcctgattacaagaaactgagcttcccagagggctttaagtgggagagagtgatgaattttgaagatgggggcctggtgaccgtgacacaagacagcagcctccaggacggcactctgatctacaaagtcaaaatgcgcggcaccaattttccacccgatggacccgtgatgcagaagaaaacaatgggatgggaggcttccactgaaaggctgtatcctagagacggggtgctgaagggcgaaatccaccaggccctgaagctgaaagacggagggcactacctggtcgagttcaagaccatctacatggccaagaaaccagtgcagctgcccggctattactatgtggacaccaagctggatatcacaagccacaatgaagactacactattgtggaacagtatgagcggtccgaagggcgccaccatctgtttctgtacggcatggatgagctgtataagACTAGTAGCGGCCGCTGCAG

>BBa_J96031 EGFP Biobrick

GAATTCGCGGCCGCTTCTAGAgtgagcaagggcgaggagctgttcaccggggtggtgcccatcctggtcgagctggacggcgacgtaaacggccacaagttcagcgtgtccggcgagggcgagggcgatgccacctacggcaagctgaccctgaagttcatctgcaccaccggcaagctgcccgtgccctggcccaccctcgtgaccaccctgacctacggcgtgcagtgcttcagccgctaccccgaccacatgaagcagcacgacttcttcaagtccgccatgcccgaaggctacgtccaggagcgcaccatcttcttcaaggacgacggcaactacaagacccgcgccgaggtgaagttcgagggcgacaccctggtgaaccgcatcgagctgaagggcatcgacttcaaggaggacggcaacatcctggggcacaagctggagtacaactacaacagccacaacgtctatatcatggccgacaagcagaagaacggcatcaaggtgaacttcaagatccgccacaacatcgaggacggcagcgtgcagctcgccgaccactaccagcagaacacccccatcggcgacggccccgtgctgctgcccgacaaccactacctgagcacccagtccgccctgagcaaagaccccaacgagaagcgcgatcacatggtcctgctggagttcgtgaccgccgccgggatcactctcggcatggacgagctgtacaagACTAGTAGCGGCCGCTGCAG

>BBa_J96032 Cerulean Biobrick

GAATTCGCGGCCGCTTCTAGAgtgagcaagggcgaggagctgtttaccggcgtggtgccgattctggtggaactggatggtgatgtgaacggccataaatttagcgtgagcggcgaaggcgaaggtgatgcgacctatggcaaactgaccctgaaattcatttgcaccaccggcaaactgccggttccgtggccgaccctggttaccaccctgacctggggcgtgcagtgctttgcgcgttatccggatcatatgaaacagcacgatttctttaaaagcgccatgccggaaggctatgtgcaggaacgcaccatcttttttaaagatgatggcaactataaaacccgtgcggaagtgaaatttgaaggcgataccctggtgaaccgtattgaactgaaaggcatcgatttcaaagaagatggcaacattctgggccataaactggaatataactacatcagcgataacgtgtatatcaccgcggataaacagaaaaacggcatcaaagcgaactttaaaatccgccacaacattgaagatggcagcgtgcagctggccgatcattatcagcagaacaccccgattggtgatggcccggtgctgctgccggataaccattatctgagcacccagagcaaactgagcaaagatccgaacgaaaaacgtgatcacatggtgctgctggaatttgtgaccgcagccggtattaccctgggcatggacgagctgtacaagACTAGTAGCGGCCGCTGCAG

>BBa_J96033 EBFP2 Biobrick

GAATTCGCGGCCGCTTCTAGAgtgagcaagggcgaggagctgttcaccggggtggtgcccatcctggtcgagctggacggcgacgtaaacggccacaagttcagcgtgaggggcgagggcgagggcgatgccaccaacggcaagctgaccctgaagttcatctgcaccaccggcaagctgcccgtgccctggcccaccctcgtgaccaccctgagccacggcgtgcagtgcttcgcccgctaccccgaccacatgaagcagcacgacttcttcaagtccgccatgcccgaaggctacgtccaggagcgcaccatcttcttcaaggacgacggcacctacaagacccgcgccgaggtgaagttcgagggcgacaccctggtgaaccgcatcgagctgaagggcgtcgacttcaaggaggacggcaacatcctggggcacaagctggagtacaacttcaacagccacaacatctatatcatggccgtcaagcagaagaacggcatcaaggtgaacttcaagatccgccacaacgtggaggacggcagcgtgcagctcgccgaccactaccagcagaacacccccatcggcgacggccccgtgctgctgcccgacagccactacctgagcacccagtccgtgctgagcaaagaccccaacgagaagcgcgatcacatggtcctgctggagttccgcaccgccgccgggatcactctcggcatggacgagctgtacaagACTAGTAGCGGCCGCTGCAG

>BBa_J63000 mCherry Biobrick

GAATTCGCGGCCGCTTCTAGAgtgagcaaaggcgaggaagataacatggcgatcattaaagaatttatgcgctttaaagtgcatatggaaggcagcgtgaacggccatgaatttgaaattgaaggcgaaggcgaaggtcgtccgtatgaaggcacccagaccgcgaaactgaaagtgaccaaaggcggtccgctgccgtttgcgtgggatattctgagcccgcagtttatgtatggcagcaaagcgtatgtgaaacatccggcggatattccggattatctgaaactgagctttccggaaggctttaaatgggaacgcgtgatgaactttgaagatggcggcgtggtgaccgtgacccaggatagcagcctgcaagatggcgaatttatctataaagtgaaactgcgtggcaccaactttccgagcgatggcccggtgatgcagaaaaaaaccatgggctgggaagcgagcagcgaacgtatgtatccggaagatggcgcgctgaaaggcgaaattaaacagcgcctgaaactgaaagatggcggccattatgatgcggaagtgaaaaccacctataaagcgaaaaaaccagtgcagctgccgggtgcgtataacgtgaacatcaaactggatatcaccagccacaacgaagattataccatcgtggaacagtatgaacgtgcggaaggccgtcatagcaccggcggcatggatgaactgtataaaACTAGTAGCGGCCGCTGCAG

>BBa_J96034 RLuciferase Biobrick

GAATTCGCGGCCGCTTCTAGAgcttccaaggtgtacgaccccgagcaacgcaaacgcatgatcactgggcctcagtggtgggctcgctgcaagcaaatgaacgtgctggactccttcatcaactactatgattccgagaagcacgccgagaacgccgtgatttttctgcatggtaacgctgcctccagctacctgtggaggcacgtcgtgcctcacatcgagcccgtggctagatgcatcatccctgatctgatcggaatgggtaagtccggcaagagcgggaatggctcatatcgcctcctggatcactacaagtacctcaccgcttggttcgagctgctgaaccttccaaagaaaatcatctttgtgggccacgactggggggcttgtctggcctttcactactcctacgagcaccaagacaagatcaaggccatcgtccatgctgagagtgtcgtggacgtgatcgagtcctgggacgagtggcctgacatcgaggaggatatcgccctgatcaagagcgaagagggcgagaaaatggtgcttgagaataacttcttcgtcgagaccatgctcccaagcaagatcatgcggaaactggagcctgaggagttcgctgcctacctggagccattcaaggagaagggcgaggttagacggcctaccctctcctggcctcgcgagatccctctcgttaagggaggcaagcccgacgtcgtccagattgtccgcaactacaacgcctaccttcgggccagcgacgatctgcctaagatgttcatcgagtccgaccctgggttcttttccaacgctattgtcgagggagctaagaagttccctaacaccgagttcgtgaaggtgaagggcctccacttcagccaggaggacgctccagatgaaatgggtaagtacatcaagagcttcgtggagcgcgtgctgaagaacgagcagACTAGTAGCGGCCGCTGCAG

>BBa_J96035 Flag Biobrick

GAATTCGCGGCCGCTTCTAGAgattataaagatgatgatgataaaACTAGTAGCGGCCGCTGCAG

>BBa_J96036 HA Biobrick

GAATTCGCGGCCGCTTCTAGAtatccatatgatgttccagattatgctACTAGTAGCGGCCGCTGCAG

>BBa_J96037 His Biobrick

GAATTCGCGGCCGCTTCTAGAcatcatcatcatcatcatACTAGTAGCGGCCGCTGCAG

>BBa_J96038 StrepII Biobrick

GAATTCGCGGCCGCTTCTAGAtggagccacccgcagttcgaaaagACTAGTAGCGGCCGCTGCAG

>BBa_J96014 SP Biobrick

GAATTCGCGGCCGCTTCTAGAgccaccatggagacagacacactcctgctatgggtactgctgctctgggttccaggttccactggtgacACTAGTAGCGGCCGCTGCAG

>BBa_J96015 TMD Biobrick

GAATTCGCGGCCGCTTCTAGAgctgtgggccaggacacgcaggaggtcatcgtggtgccacactccttgccctttaaggtggtggtgatctcagccatcctggccctggtggtgctcaccatcatctcccttatcatcctcatcatgctttggcagaagaagccacgtACTAGTAGCGGCCGCTGCAG

>BBa_J96016 Myristoylation Biobrick

GAATTCGCGGCCGCTTCTAGAgccaccatggggagtagcaagagcaagcctaaggaccccagccagcgcACTAGTAGCGGCCGCTGCAG

>BBa_J63008 NLS Biobrick

GAATTCGCGGCCGCTTCTAGAcccaagaaaaagcgcaaggtaACTAGTAGCGGCCGCTGCAG

>BBa_J96017 Loxp Biobrick

GAATTCGCGGCCGCTTCTAGAataacttcgtataatgtatgctatacgaagttatgcACTAGTAGCGGCCGCTGCAG

>BBa_J96018 Lox66 Biobrick

GAATTCGCGGCCGCTTCTAGAataacttcgtatagcatacattatacgaacggtagcACTAGTAGCGGCCGCTGCAG

>BBa_J96019 Lox71 Biobrick

GAATTCGCGGCCGCTTCTAGAtaccgttcgtatagcatacattatacgaagttatccACTAGTAGCGGCCGCTGCAG

>BBa_J96040 IRES Biobrick

GAATTCGCGGCCGCTTCTAGAcccccccccctaacgttactggccgaagccgcttggaataaggccggtgtgcgtttgtctatatgttattttccaccatattgccgtcttttggcaatgtgagggcccggaaacctggccctgtcttcttgacgagcattcctaggggtctttcccctctcgccaaaggaatgcaaggtctgttgaatgtcgtgaaggaagcagttcctctggaagcttcttgaagacaaacaacgtctgtagcgaccctttgcaggcagcggaaccccccacctggcgacaggtgcctctgcggccaaaagccacgtgtataagatacacctgcaaaggcggcacaaccccagtgccacgttgtgagttggatagttgtggaaagagtcaaatggctctcctcaagcgtattcaacaaggggctgaaggatgcccagaaggtaccccattgtatgggatctgatctggggcctcggtgcacatgctttacatgtgtttagtcgaggttaaaaaACTAGTAGCGGCCGCTGCAG

>BBa_J96041 P2A Biobrick

GAATTCGCGGCCGCTTCTAGAgccacgaagcaagcaggagatgttgaagaaaaccccgggcctACTAGTAGCGGCCGCTGCAG

>BBa_J96042 T2A Biobrick

GAATTCGCGGCCGCTTCTAGAgagggcagaggaagtcttctaacatgcggtgacgtggaggagaatcccggccctACTAGTAGCGGCCGCTGCAG

>BBa_J96043 PS3 Biobrick

GAATTCGCGGCCGCTTCTAGAtgacaaactgtacatgccgttaactgtaattttgcgtgatttttttgtagACTAGTAGCGGCCGCTGCAG

>BBa_J96044 PS4 Biobrick

GAATTCGCGGCCGCTTCTAGAaggtggtagccgcaaacatagttcaatacaaacttgctgtctcggcggACTAGTAGCGGCCGCTGCAG

>BBa_J96020 Linker Biobrick

GAATTCGCGGCCGCTTCTAGAaccggcggcagcgagcgccccctgaccggcggcagcgagcgccccctgaccggcggcagcgagcgccccctgACTAGTAGCGGCCGCTGCAG

>BBa_J96021 Spacer1 Biobrick

GAATTCGCGGCCGCTTCTAGAgaggttctgttaagtaactgaacccaatgtcgttagtgacgcttacctcttaagaggtcactgacctaacatACTAGTAGCGGCCGCTGCAG

>BBa_J96022 Spacer2 Biobrick

GAATTCGCGGCCGCTTCTAGAttcctccagagcgataagtgcgtgttgtctattcaacttcgagctggtacggttacaatcgccgtgagacACTAGTAGCGGCCGCTGCAG

>BBa_J96023 MS2 Biobrick

GAATTCGCGGCCGCTTCTAGAgcttctaactttactcagttcgttctcgtcgacaatggcggaactggcgacgtgactgtcgccccaagcaacttcgctaacggggtcgctgaatggatcagctctaactcgcgatcacaggcttacaaagtaacctgtagcgttcgtcagagctctgcgcagaatcgcaaatacaccatcaaagtcgaggtgcctaaagtggcaacccagactgttggtggtgaagagcttcctgtagccggatggagatcttacttaaatatggaactaaccattccaattttcgctacgaactccgactgcgagcttattgttaaggcaatgcaaggtctcctaaaagatggaaacccgattccctcggccatcgcagcaaactccggcatctacACTAGTAGCGGCCGCTGCAG

>BBa_J96024 MS2BS Biobrick

GAATTCGCGGCCGCTTCTAGAaaacatgaggatcacccatgtcagctggtcgacctgcttACTAGTAGCGGCCGCTGCAG

>BBa_J96025 LambdaN Biobrick

GAATTCGCGGCCGCTTCTAGAatggacgcccagacccgccgccgcgagcgccgcgccgagaagcaggcccagtggaaggccgccaacACTAGTAGCGGCCGCTGCAG

>BBa_J96026 BoxB Biobrick

GAATTCGCGGCCGCTTCTAGAagggcccttcttcagggccctACTAGTAGCGGCCGCTGCAG

>BBa_J96027 TEVSite Biobrick

GAATTCGCGGCCGCTTCTAGAgaaaacctgtattttcagggcACTAGTAGCGGCCGCTGCAG

>BBa_96046 d1PEST Biobrick

GAATTCGCGGCCGCTTCTAGAaagcttagccatggcttcccgccggcggtggcggcgcaggatgatggcacgctgcccatgtcttgtgcccaggagagcgggatggaccgtcaccctgcagcctgtgcttctgctaggatcaatgtgtagACTAGTAGCGGCCGCTGCAG

>BBa_96047 d2PEST Biobrick

GAATTCGCGGCCGCTTCTAGAaaacttagccatggcttcccgccggaggtggaggagcaggatgatggcacgctgcccatgtcttgtgcccaggagagcgggatggaccgtcaccctgccgcctgtgcttctgctaggatcaatgtgtagACTAGTAGCGGCCGCTGCAG
